# Telehealth Interventions Delivering Home-based Support Group Videoconferencing: Systematic Review

**DOI:** 10.2196/jmir.8090

**Published:** 2018-02-02

**Authors:** Annie Banbury, Susan Nancarrow, Jared Dart, Leonard Gray, Lynne Parkinson

**Affiliations:** ^1^ School of Nursing, Midwifery and Social Sciences Central Queensland University Rockhampton Australia; ^2^ School of Health and Human Sciences Southern Cross University Lismore Australia; ^3^ Office of the Deputy Vice Chancellor (Research) Southern Cross University Lismore Australia; ^4^ Faculty of Health Sciences Bond University Gold Coast Australia; ^5^ Centre for Health Services Research The University of Queensland Brisbane Australia; ^6^ School of Medicine and Public Health Newcastle University Newcastle Australia

**Keywords:** videoconferencing, telemedicine, patient education as topic, social support, review

## Abstract

**Background:**

Group therapy and education and support sessions are used within health care across a range of disciplines such as chronic disease self-management and psychotherapy interventions. However, there are barriers that constrain group attendance, such as mobility, time, and distance. Using videoconferencing may overcome known barriers and improve the accessibility of group-based interventions.

**Objective:**

The aim of this study was to review the literature to determine the feasibility, acceptability, effectiveness, and implementation of health professional–led group videoconferencing to provide education or social support or both, into the home setting.

**Methods:**

Electronic databases were searched using predefined search terms for primary interventions for patient education and/or social support. The quality of studies was assessed using the Mixed Methods Appraisal Tool. We developed an analysis framework using hierarchical terms feasibility, acceptability, effectiveness, and implementation, which were informed by subheadings.

**Results:**

Of the 1634 records identified, 17 were included in this review. Home-based groups by videoconferencing are feasible even for those with limited digital literacy. Overall acceptability was high with access from the home highly valued and little concern of privacy issues. Some participants reported preferring face-to-face groups. Good information technology (IT) support and training is required for facilitators and participants. Communication can be adapted for the Web environment and would be enhanced by clear communication strategies and protocols. A range of improved outcomes were reported but because of the heterogeneity of studies, comparison of these across studies was not possible. There was a trend for improvement in mental health outcomes. Benefits highlighted in the qualitative data included engaging with others with similar problems; improved accessibility to groups; and development of health knowledge, insights, and skills. Videoconference groups were able to replicate group processes such as bonding and cohesiveness. Similar outcomes were reported for those comparing face-to-face groups and videoconference groups.

**Conclusions:**

Groups delivered by videoconference are feasible and potentially can improve the accessibility of group interventions. This may be particularly useful for those who live in rural areas, have limited mobility, are socially isolated, or fear meeting new people. Outcomes are similar to in-person groups, but future research on facilitation process in videoconferencing-mediated groups and large-scale studies are required to develop the evidence base.

## Introduction

Group work is commonly used within health care across a range of disciplines such as chronic disease self-management (CDSM) and to provide psychotherapy, education, and group support. Groups are beneficial as they provide opportunities to meet others with similar health issues or in similar circumstances, learn from peers, develop self-awareness, give and receive feedback, and recognize that others share comparable challenges that can lead to more success with self-management [1]. Within the field of psychotherapy, group treatment provides crucial therapeutic elements such as universality, group cohesiveness, and interpersonal learning, all of which promote positive individual outcomes [2].

However, there are a number of barriers for participants to attending groups. Reasons for nonparticipation include mobility-reducing physical health issues, time constraints, distance, insufficient funds, lack of respite care if caring for someone else, and transportation [3]. From an organizational perspective, groups enable scarce resources to be used effectively. For instance, diabetes education often uses group settings to reduce the pressure on health staff resources given the increasing numbers of people diagnosed with diabetes [4]. Using home-based videoconferencing may be one opportunity to reduce these known barriers and improve the accessibility of group-based interventions.

Web-based groups, commonly called online groups, are used for health professional and peer-led health education and social support [5-7] and in behavior change interventions [8,9]. Online support groups can be asynchronous or synchronous, providing a range of therapeutic benefits that are similar to face-to-face support groups [10,11], and online education and behavior change interventions have reported improvements in health outcomes [9,12]. However, a systematic review on the effectiveness of online health behavior change interventions concluded that although most studies report improvements, effect sizes range widely and were generally small in magnitude [8].

Most online groups have been text-based, using discussion boards; few have used videoconferencing. Although videoconferencing has been used in a range of medical disciplines, it is still not widely adopted, and the research focus to date has been on using videoconferencing for individual patient consultations [13]. Those studies that have used group videoconferencing have employed differing configurations and technology such as all participants located at either one site or several participating sites (often a community health center) and the facilitator or facilitators located at another site [14-17]. Other formats for group videoconferencing interventions include mixing face-to-face meetings and group videoconferences [18], enabling participants to hear each other but not see each other [19], and using virtual environments for groups [20].

There have been concerns regarding the effectiveness of videoconferencing groups, which may have deterred uptake of this technology. A key outcome for using groups in health care is the social support that can be fostered by members. Some have argued that social interaction may be lacking in Internet-based programs [21], and the convenience of increased access has the potential to reduce engagement within videoconferencing groups. Compared with in-person participation, videoconferencing groups may feel artificial, disconnection with others, and engender privacy concerns [22].

Few studies have used videoconferencing to deliver group-based education [23]. It has been more widely used in psychological interventions. A review containing two studies concluded that conducting group therapy by videoconferencing is as feasible and effective as an in-person group and that technology increased access to services but did not forgo the change mechanisms in group therapy [24]. However, no previous reviews have identified factors affecting implementation and outcomes of group-based education by videoconferencing. The aim of this study was to undertake a systematic review of the literature to determine the feasibility, acceptability, effectiveness, and implementation of health professional-led group videoconferencing to provide education and/or social support into the home setting.

## Methods

### Literature Search

Publications were collected from January 2000 to March 2016 on videoconferencing group education and/or social support into the home between health professionals and groups of patients or consumers. The following electronic databases were searched: Academic Search, CINAHL with full-text, Health Source Consumer, Health Source Nursing, MEDLINE, Psychology and Behavioural Sciences Collection, PsycINFO, SocioIndex, PubMed, InfoRMIT, ProQuest, and Google Scholar. Databases included literature that was peer-reviewed and gray literature. Table 1 provides the search terms that were tailored according to the database. Search terms were identified from initial literature scoping and not restricted to the title only.

**Table 1:** Search terms. The symbol ∗ denotes truncation in the search. MeSH: Medical Subject Headings.

Step in search strategy	Search term
1	Telemedicine [MeSH] OR telecare OR telemonitoring OR telehomecare OR internet-based care/programs OR virtual OR web-based OR multi-site OR multisite
2	Videoconferencing [MeSH term] OR real-time OR synchronous
3	Health literacy [MeSH term] OR chronic disease self-management OR self-care [MeSH term] OR patient education as topic [Mesh term] OR health education [MeSH term] OR educat* OR train* OR social support [MeSH term] OR therap* OR life style [MeSH term] OR peer support OR peer educat* OR telerehabilitation [Mesh term]
4	Feasibility Studies [Mesh term] OR feasibil* OR Patient Satisfaction [MeSH term] OR accept* OR Program Evaluation [MeSH term] OR effective*
5	Adults
6	Limits: English Language; abstract; publication date January 2000 to March 2016
7	1 and 2 and 5 and 6
8	1 and 2 and 3 and 5 and 6
9	1 and 2 and 3 and 4 and 5 and 6

**Figure 1:**
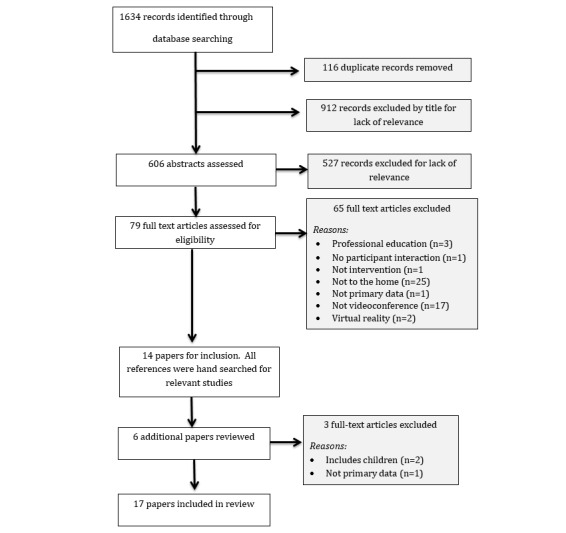
Study selection flow diagram.

### Study Selection

Included studies were interventions that collected primary data directly from participants, which documented the use of group videoconferencing for patient education or social or mental health support into participants’ homes. Intervention studies that were delivered by family practice, local primary care organizations, generalist community health services (including home nursing, counseling, allied health, and health education) and tertiary settings to the community to adults aged 18 years or older were included. Excluded studies were those that provided group education to youth or children, students, health professionals, were part of a virtual reality game, or did not enable participants to see and/or hear others in the group. No restrictions were imposed on the quality of the literature because initial assessment suggested there was a limited number of interventions conducted using group videoconferencing. In particular, studies that have delivered group videoconferencing into the home as opposed to a community health care setting are less common.

The Preferred Reporting Items for Systematic Reviews and Meta-Analyses (PRISMA) [25] flowchart representing the study selection process is shown in Figure 1. Potential eligible studies were identified by author AB scanning all 1634 titles. Authors AB and LP independently conducted an abstract review of the 606 remaining studies followed by a full-text review of 79 studies for final inclusion. Hand reference searching of the 14 remaining studies identified 6 additional studies for full-text review, of which 3 were excluded. Where there was uncertainty about potential eligibility, the third author SN read the paper, enabling a decision to be made. In total, 17 studies were included in the review. Table 2 provides results of database searches.

**Table 2:** Quality assessment of studies.

Author, year	Strength of evidence	Main features
Adamski, 2009 [31]	Low	Mixed-methods comparison study, method of qualitative data gathering is unclear, analysis unclear, no detail on quantitative data for comparison or intervention group
Austrom, 2015 [32]	Low	Mixed-methods prospective cohort pilot study, no control group, small numbers (n=4), no details on analysis for qualitative data, integration of data limited
Banbury, 2014 [33]	High	Qualitative study using three evaluation methods, satisfactory numbers (n=52), method of analysis reported
Burkow, 2013 [34]	High	Qualitative study using interviews, sample selection unclear, analysis clear, intervention well described
Burkow, 2015 [35]	High	Mixed-methods prospective cohort study, no control group, small sample size (n=10), qualitative data from interviews, findings well integrated
Damianakis, 2016 [36]	High	Qualitative study using archived recordings of videoconference meetings, content analysis and criteria well reported, three authors independently coding
Ehlers, 2015 [37]	Low	Mixed-methods randomized controlled study using two comparison groups, recruitment and randomization unclear, small numbers (n=30), qualitative data from interviews, field notes and journal, three researchers independently coding, limited integration
Khatri, 2014 [38]	High	Mixed-methods cohort prospective pilot study, small numbers (n=18), two comparison groups, qualitative data from transcripts of group meetings, two researchers independently coding, data well integrated
Lundberg, 2014 [39]	Low	Qualitative case study, interviews, field notes, and website data; methods of meetings unclear; analysis unclear
Marziali, 2006a and 2006b [40,41]	Low	Mixed-methods randomized controlled study, randomization unclear, outcome data for <80% of participants, qualitative data from archived video sessions, analysis clear
Marziali, 2009 [42]	High	Qualitative study, archived videoconference recordings and interviews, analysis clear, small size (n=18)
Marziali, 2011 [43]	High	Mixed-methods comparison study; qualitative data archived from videoconference meetings, chat sessions, and interviews; size satisfactory (n=91); two independent coders; good integration of data
Nyström, 2006 and 2008 [44,45]	High	Qualitative study, diary notes, and interviews; researcher as observer but not considered in findings
Tsaousides, 2014 [46]	Low	Mixed-methods cohort nonrandomized prospective study, no control group, small number (n=7), outcome data for >80% of measures, bias sample
Wild, 2015 [47]	High	Quantitative randomized controlled study, satisfactory numbers (n=117), clear randomization

**Table 3:** Analysis framework definitions.

Overarching theme	Definition
Feasibility	Feasibility tests the viability of the study to see whether the study can be performed [49]. For this study, it focuses on the installation and testing of equipment [52]. It includes factors relating to the videoconferencing system, equipment, and its usability for participants and facilitators. It encompasses understanding what technology factors hindered or helped with connecting groups of people and enabling facilitation and discussion
Acceptability	Acceptability relates to the extent to which the intervention is suitable, satisfying, or attractive to the participants [53]. Issues influencing acceptability included feelings of intrusiveness and invasion of privacy; whether improved exposure was beneficial, such as connecting with new people in similar circumstances; participants and facilitators ability to adapt their communication for the videoconferencing environment; attendance and dropout rates; and length of intervention
Effectiveness	Effectiveness concerns the interventions effect on participants’ health status and/or health outcomes [51,54]. Effectiveness incorporates data on whether the intervention changed something in the person, either an attribute, or their circumstances. It includes whether the intervention enabled a successful group process demonstrating cohesion and universality. In addition, whether participants felt or received empathy toward others and changes to levels of social support, social isolation, or loneliness were extracted
Implementation	Implementation is the extent the intervention can be successfully and reliably delivered to participants as it is intended [38,51,53]. In particular, studies that sought to evaluate whether an existing face-to-face intervention could be reliably replicated using group videoconferencing were included. Data were extracted for the online group process only

### Quality Assessments of Included Studies

Quality assessment of identified studies was completed using the Mixed Methods Appraisal Tool (MMAT) [26] as 7 of the 14 included studies had used mixed-methods study designs. The MMAT has met validity and reliability standards [27], is suited to a public health context, and has been used in a number of systematic reviews that comprise studies with nonrandomized controlled trial papers [28-30]. Quality assessment was conducted independently by AB and LP, with differences of opinions discussed with SN.

### Analysis Framework

The outcome terms of feasibility, acceptability, and effectiveness were often used in the included studies, but there were no consistent definitions. Telehealth literature was reviewed first to define the concepts of these terms (Table 3). The additional overarching theme of implementation was also included to capture data regarding validity and reliability of delivering face-to-face programs in the videoconferencing context. Subheadings informing the overarching themes were inductively derived from the identified studies. These concepts were then used as the framework for data extraction (Figure 1). The framework utilizes similar concepts identified by Hebert [48], where system quality, user satisfaction, and individual impact conceptualize the structure-process-outcome of telehealth variables. Our overarching concepts are present in other models that are designed to guide planning and evaluation of telehealth interventions [49-51]. However, in our framework, we have narrowed our focus of feasibility to capture data only relating to technology factors and acceptability to comprise only of patient satisfaction subjective data, enabling greater clarity between the two concepts.

### Data Extraction and Synthesis

Using the analysis framework, data were extracted from the eligible studies into an Excel (Microsoft) spreadsheet. For mixed-methods studies, qualitative and quantitative data were extracted simultaneously. Following data extraction, the studies were split into two groups comprising high- and low-level quality assessments. Content analysis compared subheading level data of the two groups with confirming and contrasting results noted. Using two groups to compare results is intended to provide greater validity for quantitative data and trustworthiness for qualitative data [55].

A narrative synthesis of data was undertaken to summarize the findings from individual studies descriptively and focused on aggregative synthesis, bringing together evidence and looking for generalizable lessons [56]. This narrative synthesis reports descriptive themes on successful and unsuccessful factors for delivering group videoconferences into the home, regardless of the topic or subject of the group work.

## Results

### Study Selection

We identified 1634 studies from the selected databases (see Table 4).

**Figure 2:**
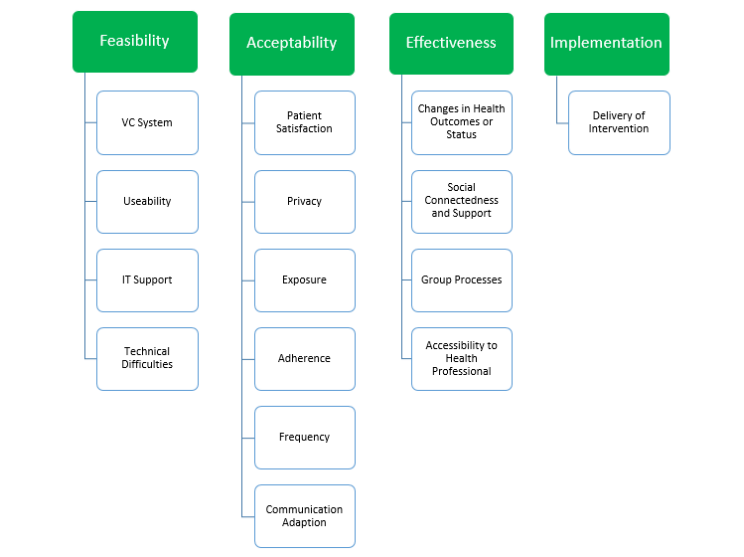
Analysis framework. VC: videoconferencing; IT: information technology.

Figure 2 provides a summary of the study selection method. Two studies were reported in four papers, and in accordance with MMAT guidelines, only one MMAT was completed for each of those studies [40,41,44,45]. Multimedia Appendix 1 provides details of the level of evidence and key factors influencing the decision-making process. There were 9 high-quality studies and 6 of low quality. A common feature of low-quality studies was the use of mixed-methods with small sample sizes and limited detail on the method of integration of quantitative and qualitative data [31,32,37,40,46].

### General Study Characteristics

Table 5 provides a summary of the included studies. There were 17 publications: five were from Canada [36,38,40-43], four from the United States [31,32,37,46], two from Sweden [39,44,45], two from Norway [34,35], and one each from Australia [33] and Germany [47]. They included 14 observational studies and three randomized control trials [37,40,47]. Sample sizes ranged from 4 to 117. Of the included studies, 9 were mixed, 6 were qualitative, and 1 used quantitative methods.

**Table 4:** Number of studies retrieved from databases.

Database	Number of studies retrieved
PubMed	951
Academic Search, CINAHL with full text, Health Source Consumer, Health Source Nursing, MEDLINE, Psychology and Behavioural Sciences Collection, PsycINFO, SocioIndex	246
InfoRMIT	45
ProQuest-narrow and refined terms	45
Google Scholar	344
Reference searching	3

**Table 5:** General study characteristics of included studies.

Author, year, country	Aim of study	Type of group; group lead	Methodology	Full^a^ or part^b^	Level of evidence
Adamski, 2009, United States [31]	Support to caregivers of persons with dementia	Psychoeducational; Unspecified	Mixed	Full	Low
Austrom, 2015, United States [32]	Support to caregivers of persons with dementia	Psychoeducational;Psychologist	Mixed	Full	Low
Banbury, 2014, Australia [33]	Health literacy and chronic disease education	Educational; Health Promotion Specialist	Qualitative	Full	High
Burkow, 2013, Norway [34]	Pulmonary rehabilitation and diabetes education	Educational and exercise; Multidisciplinary	Qualitative	Part	High
Burkow, 2015, Norway [35]	Pulmonary rehabilitation	Educational and exercise; Multidisciplinary	Mixed	Full	High
Damianakis, 2016, Canada [36]	Support to caregivers of survivors of traumatic brain injury	Psychoeducational; Social worker	Qualitative	Part	High
Ehlers, 2015, United States [37]	Book club to improve physical activity behaviors	Educational; Health Promotion Specialist	Mixed	Part	Low
Khatri, 2014, Canada [38]	Cognitive behavioral therapy	Psychoeducational; Nurse	Mixed	Part	High
Lundberg, 2014, Sweden [39]	Support for caregivers of persons with dementia or stroke survivor	Educational; Nurse and social worker	Qualitative	Part	Low
Marziali, 2006a and 2006b, Canada [40,41]	Support for caregivers with neurodegenerative disease	Psychoeducational; Social worker and nurse	Mixed	Part	Low
Marziali, 2009, Canada [42]	Healthy lifestyles program for persons with chronic disease	Educational; Not specified	Qualitative	Part	High
Marziali, 2011, Canada [43]	Support caregivers of persons with dementia	Psychoeducational; Nurses and social workers	Mixed	Part	High
Nyström, 2006 and 2008, Sweden [44,45]	Support for new parents	Facilitated support; Child Health Nurse	Qualitative	Full	High
Tsaousides, 2014, United States [46]	Cognitive behavioral therapy treatment for emotion regulation for persons with traumatic brain injury	Psychoeducational; Psychotherapist	Mixed	Full	Low
Wild, 2015, Germany [47]	Weight loss education for persons following bariatric surgery	Psychoeducational; Psychotherapist	Quantitative	Part	High

^a^Intervention only comprised videoconference groups.

^b^Intervention comprised other elements such as online education.

### Intervention Characteristics

Multimedia Appendix 2 provides intervention characteristics of the studies. A total of 467 participants contributed to the 15 studies. Six studies targeted caregivers [31,32,36,39,40,41,43], the most predominant target group within the review. Others targeted people with chronic disease [33-35,38,42], obesity [47], traumatic brain injury [46], new parents [44,45], and those not reaching public health healthy lifestyle guidelines [37]. Eight studies reported participants’ age or average age as above 50 years, and of these, 5 participants had an average age of above 65 years, many of whom were inexperienced computer users.

The services provided by group videoconferencing were: psychoeducational [31,32,36,38,40,41,43,46,47], where the intervention included a psychological intervention or psychological-based support; therapeutic support group [44,45], where groups of people facing similar issues were brought together—these emphasize emotional support and shared experiences as participants can direct the topic and format of the group discussions, and they may also contain an educational element; and an educational support group [33-35,37,39,42] in which the groups received education and took part in facilitated discussion on specific conditions or diseases.

In 7 studies, videoconference group meetings were the only component of the intervention, whereas for the other 10 studies, the videoconference group meetings were one of multiple components. These other components included: access to information on an intervention-specific website (6); text-based discussion forums (5); email link to other participants (4); face-to-face group meetings (4); link for one-to-one health consultations with a health professional (2); link for one-to-one videoconferencing social meeting (1); and an electronic health diary for wireless transmission or manual entry of sensor data (1). In 2 studies, weekly videoconference group exercise sessions took place.

There was a range of health professionals providing group facilitation, including a specialist, psychologists, psychotherapists, social workers, nutritionists, nurses, and health promotion specialists.

Outcome measures varied between studies. Data relating to the health status and/or health outcomes were collected using both validated and nonvalidated measures. Validated measures were defined as those for which the authors provided an academic reference and the psychometric properties, such as the Short Form Health Survey-36 [57]. Nonvalidated measures were those developed for the specific purposes of the study [58]. The heterogeneous nature of the studies and the limited number of quantitative studies meant that a meta-analysis of quantitative data was inappropriate [59]. Five studies measured perceived health and health-related quality of life (HRQoL) [32,35,40,43,47], 5 depression [32,38,40,43,47], 3 social support [37,40,43], 2 caregiver self-efficacy [32,43], and 1 study measured caregiver burden [32]. Other studies explored the following factors: physical activity, general self-worth, physical self-worth, physical activity self-efficacy, physical activity self-regulation, physical activity benefits or barriers [37], activities of daily living [40], neuroticism [43], weight and eating behavior [47], emotional regulation and problem solving [46], health service use [43], and technology usability [35].

All studies included results on feasibility, acceptability, and effectiveness, and some reported issues connected with implementation [33,36-38,40-44,47].

### Feasibility

#### Videoconferencing Systems

Multimedia Appendix 2 provides details of the number of participants connected to the groups at one time and Multimedia Appendix 3 describes key findings of the studies. The majority of studies used desktop computers [32,39,41-45], 2 used tablet computers [33,37], and 2 used computers connected to users’ televisions [34,35]. Six studies used intervention-specific websites with videoconferencing group links embedded within them [36,38,40-45]. Three studies used the same website [36,40,41,43], one of which was an updated version [42], and 2 studies used the same videoconferencing system [34,35].

Devices and additional equipment such as webcams and headsets were generally supplied, although in 2 studies the inclusion criteria specified participants having access to a computer and broadband [45,46]. In another study, they used participants’ computers and Internet access but provided refurbished equipment for those who needed it [43]. Reported connection speeds were 200 to 400 kbps [32,34] and high-speed broadband [33].

#### Usability

Participants were not always experienced in videoconferencing or computer use. Overall, inexperience did not appear to be a major problem as the majority of studies reported that, over time, participants found the technology easy to use [32-35,40-42,46] and found videoconferencing enjoyable [42,44-46]. One study, in which a third of participants had a degree, reported that poor digital literacy may have contributed to low participation rates [37]; suggesting that education level is not necessarily associated with digital literacy. Other studies noted technology was not a barrier, with participants persisting in overcoming technical difficulties [32,42,43].

#### Information Technology Problems

Various levels of technical problems were encountered; 8 of the 15 studies reported few difficulties [31,32,34-36,38,44-46], whereas 7 reported a number of problems [33,37,39,42,43,45,47], 2 of which required substantial work hours to overcome [39,47]. The most common problem reported was audio difficulties, which included delays, dropouts, and background noise [33,37,38,44,45], followed by problems in downloading software [38,42,43].

Visual problems were reported less frequently but included poor lighting in participants’ homes [33] and too small a picture to clearly see body language [44,45]. External factors such as location, type of dwelling, and speed of connection also effected videoconferencing quality [33]. However, 3 studies reported that technical difficulties declined during the course of the intervention [33,38,46].

#### Training and Support

Training was provided to participants either face-to-face [34,35,40,43], with verbal and written instructions [41,46], or an emailed tutorial [37].

Most studies received information technology (IT) support during the videoconference group meetings either by IT specialists or facilitators that were able to troubleshoot problems. IT support was offered using a range of mechanisms including remotely accessing participants’ devices [32,33], talking participants through problems by telephone or online [31-36,38,39,44], information manuals [34,35,41,42], home visits (either at the start of the program during installation [43] or during the program [32-34]), and emailed tutorial [37]. For those studies that incurred several problems, participants felt frustrated and in one study needed reassuring that they were not at fault for the technical glitches [38]. Good technical support was considered an important element for an intervention, which could ease participants’ anxiety [31].

Brief training for group facilitators was reported in 2 studies [34,35]. Technical difficulties were frustrating for facilitators [38,39], and one study reported a challenging online environment where the facilitator could only see one participant in the active window and was unable to see other members facial expressions or body language [41].

There were no consistent differences in feasibility reported between studies of high quality and those of low quality.

### Acceptability

#### Patient Satisfaction

Overall, patient satisfaction with group videoconferencing was high. All but one study [37] reported that participants had found meeting in a videoconference group either satisfactory or a positive or very positive experience. Factors that contributed to this included being able to see and hear other group members, meeting new people in similar circumstances, sharing experiences, and being part of a group that provided social support. A few participants would have preferred to have met face-to-face rather than by videoconferencing, with one group citing, as their reasons for this preference, low social presence [37].

#### Adherence and Frequency

Overall, attendance at the videoconferencing groups was high, with few dropouts. Attendance rates ranged from 66% to 93.8%, with 6 studies reporting groups with rates of >80% [32,35,36,46,47]. Reasons for participants dropping out or nonattendance included technical problems, not liking to talk about their health, too busy, and illness. Three studies asked participants to complete homework, which included watching educational videos before the next session, completing health diaries, and practicing new skills [34,35,46]. Adherence rates were very good, with homework completed 93% of the time [46] and all participants viewing educational videos and completing health diaries [34,35].

The duration of group videoconferencing meetings ranged from 45 min to 105 min, typically lasting for 1 hour. The majority of videoconferencing groups met weekly. In some studies, participants wanted to meet for longer [34,35,46], more frequently [32,35], or expressed disappointment when the videoconferencing group finished [33]. In 2 studies, individual sessions were provided for discussion on personal issues that would not suitable for the group setting; however, the need to ask questions varied depending upon disease stability [34,35].

Videoconferencing groups were compared with other interventions including face-to-face [31,37,38], text-based chat forum [43], and usual/standard care [47]. Two studies reported similar or higher levels of participation compared with the alternative intervention [31,43]. One study found that attendance rates and participation by the videoconferencing group were lower compared with the face-to-face group [37] and that while on the videoconference, some participants were talking and doing other things such as making dinner and watching TV. Three studies provided 10 to 12 weeks of health professional facilitation, after which groups met on a self-help basis where a group member assumed the facilitation role [41-43]. For the self-help groups, one study reported attendance rates dropping from 70% when the groups were health professionally led to 50% for member-led groups [43]. Another group expressed the wish to continue as a self-help group but felt that without a leader this would be difficult [32].

#### Privacy and Exposure

Issues of privacy and seeing into each other’s homes were not reported as a problem in any studies. In one study, there needed to be prior agreement for someone else to be present in the room while the videoconferencing group was taking place, and the guest was required to be visible [35]. In addition, to closely guard privacy, the camera cover could be closed when not in use.

Conversely, viewing the participant’s home environment could increase tailored education and support. In a study of dementia caregivers, the facilitator and participants were able to see that a dementia patient was trying to leave the house repeatedly, which prompted the facilitator to provide safety education and information on local dementia safety services [32]. Another study, which provided support to family caregivers of survivors with traumatic brain injury, had additional family members join the support group intermittently. They were accepted by other participants as part of the group members’ on-going and evolving needs [36].

An unexpected challenge was the difficulty in obtaining participants’ consent forms, which were by mail. The researchers speculated that this was related to privacy issues of being able to see into participants’ home environment. The organization had specified on the consent forms that if they suspected any type of elder abuse, they were required to investigate the matter [31].

There were no clear differences in the reported outcomes for acceptability between studies of high and low quality.

#### Communication Adaption

Over time, the vast majority of participants became familiar with the technology and adapted their communication accordingly [33,36,42,46]. Overall, only a few participants felt uncomfortable using videoconferencing to communicate with others. Difficulties arose when several people talked at the same time and then stopped on hearing others and then after a pause started talking at the same time again [34]. It was acknowledged that structure and protocols are needed to optimize group communication [35]. Clear communication guidelines and protocols contributed to avoiding talking over each other. In 2 studies, this was reiterated at each session as well as highlighting the importance of confidentiality, active listening, and speaking slowly and clearly [33,47].

### Effectiveness

The effectiveness of interventions was considered in terms of changes in health outcomes, including improved health knowledge, insight, and skills; social connectedness and whether face-to-face group processes were replicated; engagement between participants; and increased access to a health professional.

#### Changes in Health Outcomes

Skills for development included cognitive behavioral therapy strategies [38], insight and coping strategies [40,43], ability to navigate the health care system [36], emotional regulation [46], disease-specific knowledge and skills [32,34,35], and health literacy [33].

The heterogeneity of the studies led to a wide range of assessment tools to report health status and health outcomes. For comparative studies, changes in pre- and postintervention results were similar for face-to-face groups [31,38] and usual care [47] but significantly better than a text-based forum [43].

In pre-post treatment scores, there was a significant change in HRQoL (*P*=.04) [35] but no significant differences in emotional regulation, problem solving [46], or physical activity and associated factors [37]. Of note is the trend of videoconferencing groups improving aspects of mental health and self-efficacy [32,38,43,47]. One high-quality study of participants with clinically significant depression at baseline reported that videoconferencing groups had significantly better HRQoL (*P*=.03) and lower depression score (*P*=.02) compared with the control group of usual care 1 year after surgery [47].

Health knowledge, insight, and skills were developed through didactic teaching methods, discussion, sharing experiences, asking and listening to questions, self-reflection, and books (available on an e-reader). Information that was available on websites was accessed at the beginning of the intervention but much less so as the intervention continued [37,39].

#### Social Connectedness and Support

Engaging with others who were experiencing similar problems was highly valued and enabled empathic connections to develop. High-quality studies consistently reported positive outcomes of engagement. Videoconferencing groups helped reduced feelings of anxiety, isolation, and loneliness [36,39,42,44] and provided emotional and social support [32-35]; however, some participants took a while before they felt at ease with others, which may have been related to the online environment [46]. Two studies combined face-to-face meetings with online meetings [34,39]; one study reported that, for those who could not attend the face-to-face meeting, engagement with others during the intervention was not compromised [34]. Only one study reported that the videoconferencing environment limited participants’ connection with each other [37]. Videoconferencing groups were considered superior in comparison with a text-based forum, with few people contributing to the forum and threaded discussions going off-topic [43].

#### Group Processes

Bonding and cohesiveness were reported in all high-quality studies and in one low-quality study [40,41]. Higher levels of cohesiveness were demonstrated in groups with more stable memberships compared with groups whose membership altered because of changes in participants’ availability [33]. Gender differences were noted in a study comprising one group of men and one of women, with the men’s discussions being more problem-focused and the women’s being more emotion-focused [44,45]. Qualitative studies reported discussion themes that illustrated participants’ ability to discuss sensitive and personal issues and to give and receive empathetic support [33,36,42,44,45].

#### Accessibility of Groups

Accessing a group from home was considered beneficial in all studies except one, whose participants would have preferred to have met face-to-face [37]. The ability to meet from one’s home was viewed positively and helped overcome a number of barriers that, for some participants, would have prohibited their attendance at a face-to-face group. Barriers included illness, transportation difficulties, not being able to leave the person they were caring for, and/or living rurally or in an area where there was no face-to-face alternative. Additionally, some participants reported feeling more relaxed and open by being at home and valued the convenience [33,34,36,42-46].

### Implementation

Treatment reliability and validity was assessed in four psychoeducational studies [36,38,41,43]. They aimed to demonstrate that technology-supported groups met the same standards and outcomes as face-to-face groups. The face-to-face group format and process was replicated in videoconferencing groups in 3 studies [36,38,41], and treatment protocol was adhered to in videoconferencing format in 2 of the studies [38,43]. Validity was demonstrated through the analysis of discussion themes such as cohesiveness, empathic support, problem solving, or issues in disease-specific caregiver literature and was consistent with the face-to-face groups [36,38,40-42]. Results were reported as similar to face-to-face groups [38,43,47]. In 2 studies, facilitators reported that implementing the intervention by videoconferencing was initially challenging, but over time, techniques were mastered, and the operation became more automatic [38,41]. Difficulty in retrieving online assessments and evaluation forms were reported [31,37].

Details on pre-program procedures overall were lacking but included participants being required to be ready up to 15 min before the start of the meeting, which could be used for informal chat time [33], enabling a socialization opportunity [34]; the importance of punctuality [47] and pre-program face-to-face meetings are not necessary [35].

## Discussion

### Principal Findings

We reviewed evidence of feasibility, acceptability, effectiveness, and implementation of health professional-led group videoconferencing to provide education and/or social support into the home setting. Fifteen studies met our inclusion criteria. Overall, evidence indicated that group videoconferencing into the home was feasible and acceptable, but it was harder to draw firm conclusions on the effectiveness of such interventions.

The routine and widespread use of home-based videoconferencing groups for health support applicability has as yet not been widely researched. Therefore, intervention studies identified to inform this systematic review were mostly pilot in nature and contained small sample sizes and generally were nonrandomized study types. The identified studies were considerably divergent in regards to the interventions, comparison groups, and outcome measures used. A wide range of health outcome measures were employed; however, their usefulness is debatable as sample sizes were commonly small, and therefore, studies may have been underpowered, with the quantitative data providing no new information. Overall, qualitative data provided a deeper understanding of equipment usability, IT support, privacy and exposure issues, group dynamics, and perceived benefits.

### Feasibility

Videoconferencing systems were most commonly used with desktop computers, which most studies provided for the participants. Mobile health (mHealth) devices such as tablet computers and mobile phones were infrequently used, despite their ability to provide access to videoconferencing with few technical skills. For those with limited experience in using technology, mHealth and apps can provide simplified access by overcoming difficulties such as downloading software and using a mouse. As ownership of mobile devices and access to the Internet grows, it is feasible that health programs can be developed so that participants can “bring your own devices,” as has been implemented in the education sector [60,61]. Using consumers own devices would lower program costs; however, further work in understanding issues of interoperability, security, and acceptability is warranted to investigate the use of personal devices for health care.

Good IT support was a vital component in the feasibility of delivering the interventions. The majority of studies reported few technical problems, and for those that did report difficulties, audio lag was the most common issue. IT support was mostly available during the videoconferencing groups by IT personnel or in a few studies by the facilitator, with a range of strategies used, including remote access to devices and verbal instructions. IT support is a key resource consideration for organizations proposing to use group videoconferencing interventions with clients. It is central to successful implementation for both facilitators and clients and should be adequately costed into program budgets.

The review includes studies implemented from 2006 to 2015. During this time, there has been a rapid and dramatic improvement in technology. However, later studies did not report fewer technical difficulties compared with earlier ones, but interestingly, as interventions progressed, IT problems declined. It is unclear whether this was because of participants’ technology skills improving or whether the technical problems were fixed by IT support. Geographical location and the IT systems utilized may account for technical difficulties. There were fewer technical problems reported by studies from the United States, which may pertain to more developed Internet operations and IT systems. Although IT glitches could lead to frustration, it appears that participants were persistent in overcoming difficulties, as the benefits of being part of a group and meeting others outweighed the technical difficulties.

### Acceptability

Acceptance of meeting by videoconferencing was high. Overall, participants found the experience of using videoconferencing groups positive, with few participants preferring to have met face-to-face. Some participants expressed they would have liked the programs to be more frequent or last for longer. Adherence to the programs was high, which may indicate publication bias for successful interventions. The majority of the studies targeted interventions for people aged 50 years and older, indicating, contrary to some opinions [62], technology can be used in the care of older people who may have poorer digital literacy. Inexperience in computer use did not appear to be a barrier for participants, with many studies reporting the technology was easy to use. In some populations, videoconferencing is becoming ubiquitous and a natural means of communicating. Therefore, it is not unreasonable to conclude that, in time, the use of group videoconferencing will become mainstream.

Previously, privacy issues have been cited as a barrier for telehealth implementation [62,63]. In our review, no studies reported participants concern about others seeing into their homes [64,65]. Few studies discussed the impact of interventions taking place in the home and the lack of control practitioners have in this environment. Prior consideration of delivering interventions into shared living spaces is necessary, in particular, the inclusion or exclusion of other residents. The benefits of viewing participants in their environment was highlighted, enabling education to be tailored to participants’ needs. Other studies have reported the importance of health education, taking into account the context of people’s lives [66]. Videoconferencing may provide educators with an additional understanding of contextual issues for clients, which may lead to a more patient-centered health intervention.

Few studies provided details on whether specific communication strategies were adapted to facilitate videoconferencing groups. Social presence is the extent to which a technology used to facilitate a meeting can provide a social or personable feeling to the interaction [67]. Although videoconferencing allows for a higher social presence than other computer-mediated communications such as discussion boards, it has a lower social presence compared with face-to-face meetings [68,69]. Clear communication guidelines and strategies appeared to have helped overcome some technical difficulties and aid effectiveness of the interventions [70]. However, descriptions on facilitator skills necessary for the challenging videoconferencing environment were rarely discussed. How facilitators may have changed their communication method and style would further help develop an understanding of best practice for telehealth group videoconferencing interventions. A review of videoconferencing for CDSM noted differences in attitudes between participants and health professionals, with clients more accepting of the technology [71]. These differences may be because of a more complex intervention environment for facilitators.

There is an indication that groups via videoconferencing may provide a new avenue to either kick-start new self- help groups or sustain existing groups. Although details were scant on the effectiveness or uptake, there were interventions that developed groups that were designed to continue meeting after an agreed amount of time of health professional facilitation [40,43]. Member-led self-help groups may provide a new model for cost-effective social support groups, given that, after initial set-up, there is no cost to the health service provider.

### Effectiveness

Compared with other modes of delivery, videoconferencing groups were significantly better than a text-based forum and similar to face-to-face groups and usual care. Increases in health knowledge and skills were achieved across a range of topics including mental health issues, health system use, and lifestyle behaviors. Home-based videoconferencing groups overcame known barriers for attending face-to-face groups, such as transportation, travel distance, lack of time, inconvenience [72,73], and not being able to leave the care beneficiary. However, it should be noted, as outlined earlier, there are other drawbacks such as consideration of other residents and interruptions that hinder using videoconferencing in the home environment.

A consistent finding was the perception that groups enabled engagement and social support, which was highly rated by participants. Lack of social support, social isolation, and loneliness are known risk factors for ill health and hospitalization [74,75]. Using new technology to help develop social support networks and overcome social isolation and loneliness in real-time is an emerging area [33]. Videoconferencing groups could be used to develop new and relatively low-cost interventions, particularly with at-risk groups such as those living in rural areas, with limited mobility and older people.

Identifying which groups of people are most likely to benefit from telehealth interventions is an important factor in improving the evidence base for telehealth [76]. Telehealth interventions may not be suited for all populations, and it is important to understand which groups would be best targeted, or are most responsive to, the use of group videoconferencing, to ensure that resources are used efficiently. Due to the heterogeneous nature of the studies, it is not possible to draw any firm conclusion as to whether there are specific subgroups that are particularly suited for group videoconferencing.

However, similar to studies with videoconferencing group participants located in health care centers [17,77], there is a clear trend for improving mental health outcomes such as depression, self-efficacy, stress and anxiety, and overcoming a fear of meeting new people. Furthermore, videoconferencing groups can provide sustained mental health outcomes, as demonstrated by Wild [78], with their follow-up study reporting significantly lower depression and higher self-efficacy approximately 2 years following their group videoconferencing intervention. It is possible that being in the home environment is less stressful than meeting people in-person and that meeting by videoconferencing provides a greater feeling of anonymity [79] and security and the ability to leave the group more easily.

### Implementation

Studies that implemented existing psychoeducational interventions reported good reliability and validity and were as effective as face-to-face interventions. In addition, many studies reported the ability to replicate group processes such as bonding, cohesiveness, and empathy.

We did not specifically consider cost-effectiveness in this review but of note is the potential savings that videoconferencing groups may provide. In one study, providing rehabilitation to home-based groups decreased costs by 50% compared with face-to-face outpatient rehabilitation [35]. Cost-effectiveness has been reported for face-to-face group–based approaches for CDSM programs [80], and there may be even greater cost savings if groups are delivered by videoconferencing. Cost savings to the health provider can be made by educating a number of people simultaneously, more efficient use of clinical time, and it may even reduce the numbers of nonattendance [81]. For patients, particularly those in rural areas, videoconferencing improves access to health professionals and removes time-consuming and expensive travel costs. As people age, their use of health care services increases, and therefore, an understanding of whether group videoconferencing would be acceptable and cost-effective in providing interventions to older populations who are high users of health services would be valuable. The cost-effectiveness of group videoconferencing compared with usual care may encourage uptake and is suggested as an area for further research.

### Limitations

Comparability of study findings was limited by the heterogeneity of the interventions, participants, and assessed outcomes. Sample sizes were small, which was a limitation for those studies reporting quantitative data. However, the number of studies in the field was so limited that all relevant studies to identify commonalties and consistent themes were reviewed. In addition, identifying the limitations of videoconferencing-only interventions was not possible as studies that included other elements such as face-to-face meetings or text-based discussion forums did not report separate findings.

The range of different tools used to measure the same health outcome, such as depression, meant it was not possible to compare the effectiveness of studies. Adoption of consistent tools for telehealth interventions would enable outcomes to be compared and further advance the evidence base. Telehealth is an emerging field, and new tools are likely to be developed specifically for this use. Indeed, the new Whole Systems Demonstrator Users Technology Acceptability Questionnaire measures a range of user beliefs and identifies who are more likely to refuse telehealth [82]. This tool was developed since this systematic review and may provide researchers with a consistent tool that is suitable for a range of telehealth programs.

Limiting study eligibility to health intervention videoconferencing groups delivered to the home rather than to another setting may have produced bias. During the search strategy, 25 studies were identified that delivered videoconferencing groups into health care settings. The decision to limit the search to those delivered into the home was to explore the implications for participants and facilitators in delivering home-based groups.

### Conclusions

Group videoconferences into the home are feasible but need good IT support. The benefits of being able to take part in a group from home often outweigh the frustration of IT problems. At present, interventions that have used mHealth are limited. However, it is not unreasonable to expect these to increase because of the ubiquitousness of mHealth devices. Similarly, the rapid advancement of technology suggests that technical difficulties will decrease, and there will be more interventions which experience few technical problems.

The acceptability of group videoconferencing was high in different age-related and content-related groups. Exposure into people’s homes was not a concern; in fact, it can help target interventions to be more context specific. Further work is required to identify which subgroups would benefit the most from this type of intervention, as well as understanding how to modify communication for group videoconferencing.

Group videoconferencing is effective in overcoming many barriers for accessing face-to-face groups. Evidence suggests that group processes can be replicated in the online environment. The effectiveness of interventions varied, although there was a trend to improvement for participants with mental health problems. Further research to identify which populations and the learning content most likely to benefit from group videoconferencing should be undertaken.

## Appendix

Multimedia Appendix 1 Quality assessment of studies reviewed.

Multimedia Appendix 2 Intervention characteristics of included studies.

Multimedia Appendix 3 Outcome measures and reported findings of included studies.

## Abbreviations

CDSM: chronic disease self-management
HRQoL: health related quality of life
IT: information technology
mHealth: mobile health
MMAT: Mixed Methods Appraisal Tool
