# Supplementary material for: Telehealth Interventions Delivering Home-based Support Group Videoconferencing: Systematic Review
Source: J Med Internet Res. 2018 Feb 2;20(2):e25. doi: 10.2196/jmir.8090 (PMC5816261; doi:10.2196/jmir.8090)
Supplement: Multimedia Appendix 1 [file jmir_v20i2e25_app1.pdf]

Multimedia Appendix 1. Quality assessment of studies reviewed

Author<br>Year	Strength of evidence	Main features
Adamski 2009 [45]	Low	Mixed method comparison study, method of qualitative data gathering is unclear, analysis unclear, no detail on quantitative data for comparison or intervention group
Austrom 2015 [46]	Low	Mixed method prospective cohort pilot study, no control group, small numbers (n=4), no details on analysis for qualitative data, integration of data limited
Banbury 2014 [55]	High	Qualitative study using three evaluation methods, satisfactory numbers (n=52), method of analysis reported
Burkow 2013 [53]	High	Qualitative study using interviews, sample selection unclear, analysis clear, intervention well described
Burkow 2015 [54]	High	Mixed methods prospective cohort study, no control group, small sample size (n=10), qualitative data from interviews, findings well integrated
Damianakis 2016 [49]	High	Qualitative study using achieved recordings of VC meetings, content analysis and criteria well reported, three authors independently coding
**Ehlers 2015 [47]**	Low	Mixed methods randomised controlled study using two comparison groups, recruitment and randomisation unclear, small numbers (n=30), qualitative data from interviews, field notes and journal, three researchers independently coding, limited integration
Khatri 2014 [38]	High	Mixed methods cohort prospective pilot study, small numbers (n=18), two comparison groups, qualitative data from transcripts of group meetings, two researchers independently coding, data well integrated
Lundberg 2014 [52]	Low	Qualitative case study, interviews, field notes, and website data, methods of meetings unclear, analysis unclear
Marziali 2006a & 2006b [42,41]	Low	Mixed methods randomised controlled control study, randomisation unclear, outcome data for <80% of participants, qualitative data from achieved video sessions, analysis clear
Marziali 2009 [50]	High	Qualitative study, achieved VC recordings, interviews, analysis clear, small size (n=18)

Author Year	Strength of evidence	Main features
Marziali 2011 [51]	High	Mixed methods comparison study, qualitative data achieved VC meetings chat sessions and interviews, size satisfactory (n=91), two independent coders, good integration of data
Nyström 2006 & 2008 [43,44]	High	Qualitative study, diary notes and interviews, researcher as observer but not considered in findings
Tsaousides 2014 [48]	Low	Mixed methods cohort non-randomised prospective study, no control group, small number (n=7), outcome data for >80% of measures, bias sample
Wild 2015 [56]	High	Quantitative randomised controlled study, satisfactory numbers (n=117), clear randomisation

References:

38. Khatri N, Marziali E, Tchernikov I, Shepherd N. Comparing telehealth-based and clinic-based group cognitive behavioral therapy for adults with depression and anxiety: a pilot study. Clin Interv Aging. 2014;9:765-70. doi: 10.2147/cia.s57832. PMID: 24855345.
41. Marziali E, Damianakis T, Donahue P. Internet-Based Clinical Services: Virtual Support Groups for Family Caregivers. J Technol Hum Serv. 2006b;24(2/3):39-54. doi: 10.1300/J017v24n0203. PMID: 23259160.
42. Marziali E, Donahue P. Caring for others: Internet video-conferencing group intervention for family caregivers of older adults with neurodegenerative disease. Gerontologist. 2006a;46(3):398-403. PMID: 16731880.
43. Nyström K, Öhrling K. Parental support: Mothers' experience of electronic encounters. J Telemed Telecare. 2006;12(4):194-7. PMID: 16774701.
44. Nyström K, Öhrling K. Electronic encounters: fathers' experiences of parental support. J Telemed Telecare. 2008;14(2):71-4. doi: 10.1258/jtt.2007.070605. PMID: 31214651.
45. Adamski T, Alfaro MW. Virtual psycho-educative support groups for caregivers of persons diagnosed with dementia. Caring. 2009;28(8):44-6. PMID: 19772023.
46. Austrom MG, Geros KN, Hemmerlein K, McGuire SM, Gao S, Brown SA, et al. Use of a multiparty web based videoconference support group for family caregivers: Innovative practice. Dementia. 2015;14(5):682-90. doi: 10.1177/1471301214544338. PMID: 2015-45450-009.
47. Ehlers DK, Huberty JL, de Vreede G-J. Can an Evidence-Based Book Club Intervention Delivered via a Tablet Computer Improve Physical Activity in Middle-Aged Women? Telemed J E Health. 2015;21(2):125-31. doi: 10.1089/tmj.2013.0360. PMID: 100749832.
48. Tsaousides T, D'Antonio E, Varbanova V, Spielman L. Delivering group treatment via videoconference to individuals with traumatic brain injury: a feasibility study. Neuropsychol Rehabil. 2014;24(5):784-803. doi: 10.1080/09602011.2014.907186. PMID: 24810148.

49. Damianakis T, Tough A, Marziali E, Dawson DR. Therapy Online: A Web-Based Video Support Group for Family Caregivers of Survivors With Traumatic Brain Injury. J Head Trauma Rehabil. 2016;31(4):E12-E20. PMID: 26291634.
50. Marziali E. E-health program for patients with chronic disease. Telemed E Health. 2009;15(2):176-81. PMID: 19292627.
51. Marziali E, Garcia LJ. Dementia caregivers' responses to 2 Internet-based intervention programs. Am J Alzheimers Dis Other Demen. 2011;26(1):36-43. doi: 10.1177/1533317510387586. PMID: 21282276.
53. Burkow TM, Vognild LK, Østengen G, Johnsen E, Risberg MJ, Bratvold A, et al. Internet-enabled pulmonary rehabilitation and diabetes education in group settings at home: a preliminary study of patient acceptability. BMC Med Inform Decis Mak. 2013;13(1):33. doi: 10.1186/1472-6947-13-33. PMID: 23496829.
54. Burkow TM, Vognild LK, Johnsen E, Risberg MJ, Bratvold A, Breivik E, et al. Comprehensive pulmonary rehabilitation in home-based online groups: a mixed method pilot study in COPD. BMC Res Notes. 2015;8(1):766. doi: 10.1186/s13104-015-1713-8. PMID: 26651831.
55. Banbury A, Parkinson L, Nancarrow S, Dart J, Gray L, Buckley J. Multi-site videoconferencing for home-based education of older people with chronic conditions: the Telehealth Literacy Project. J Telemed Telecare. 2014;20(7):353-9. doi: 10.1177/1357633X14552369. PMID: 99467458.
56. Wild B, Hunnemeyer K, Sauer H, Hain B, Mack I, Schellberg D, et al. A 1-year videoconferencing-based psychoeducational group intervention following bariatric surgery: results of a randomized controlled study. Surg Obes Relat Dis. 2015 Nov-Dec;11(6):1349-60. doi: 10.1016/j.soard.2015.05.018. PMID: 26421929.
