# Supplementary material for: Telehealth Interventions Delivering Home-based Support Group Videoconferencing: Systematic Review
Source: J Med Internet Res. 2018 Feb 2;20(2):e25. doi: 10.2196/jmir.8090 (PMC5816261; doi:10.2196/jmir.8090)
Supplement: Multimedia Appendix 2 [file jmir_v20i2e25_app2.pdf]

Multimedia Appendix 2. Intervention characteristics of included studies.

Author, year, country	Description of intervention	Target group, mean age (years)	Number of participants	Duration and frequency	Total VC[a] group sessions
Adamski, 2009 [45]	Education and support group comparing VC group with face-to-face group	Caregivers, 38	N=5—two groups, unknown number in each group	5 x 1 hour, frequency unspecified	Unknown
Austrom, 2015 [46]	Education (group identified needs), peer support, and guest speakers on specialized topics	Caregivers, 56.2	N=5	26 x 1 hour weekly	26
Banbury, 2014 [55]	Education on health literacy and chronic disease self-management peer support group	Persons with chronic disease, 73	N=52—nine groups, comprised 2-7 participants	6 x 45 min to 1 hour and 30 min weekly	44
Burkow, 2013 [53]	Educational program; all participants required to watch tailored education videos before sessions, complete digital diary and weekly individual	Persons with chronic obstructive pulmonary disease (COPD) and diabetes, 45-74	N=10—two groups each with 5 participants	COPD group —eight twice-weekly group sessions for 30 min and six weekly individual consultations (duration unspecified)	22

	consultations			Diabetic group—six weekly sessions and individual consultations (duration unspecified)	
Burkow, 2015 [54]	Educational and group exercise sessions. Participants required to watch educational videos, complete digital diary, pedometer and individual consultations	Persons with COPD, 45 and 74	N=10—two groups each with 5 participants	9 x 2 hour weekly: 1 hour lecture and discussion group, 30 min exercise group, and up to 15 min individual consultation	18
Damianakis, 2016 [49]	Access to website with educational information, email, text-based discussion forum, and VC link. VC group was parental training program and forum to share feelings and gain peer support	Caregivers, 19.8	N=10—two groups each with 5 participants	10 x 1 hour weekly	20

Ehlers, 2015 [47]	Theory-based book club for education and peer support group comparing face-to-face and VC groups. VC group only had access to website and pedometer steps tracker	Women not reaching physical activity guidelines, 44.77 tablet group, 48.20 face-to-face	N=30—two groups, one face-to-face and one tablet, each with 15 participants	12 x 1 hour weekly	12
Khatri, 2014 [38]	Access through website with email, text-based discussion forum, VC link, and education information. Group cognitive behavior therapy focused on socialization, basic behavior interventions, and cognitive behavior strategies	Persons with chronic disease, 50.8 VC group and 58.4 face-to-face group	N=18—two groups, 10 participants in face-to-face group, and 8 participants chose VC group	13 x 1 hour weekly	13
Lundberg, 2014 [52]	Education and support group	Caregivers, 80.2 caregivers and 72.5 care beneficiary	N=10	Unknown	Unknown
Marziali,	Website with	Caregivers,	N=66—two	10 x 1 hour	22

2006a and 2006b [42,41]	embedded link for VC groups, which delivered education, psychosocial support, and problem-solving skills	67.8	groups, control (no intervention) and intervention; and three disease-specific groups comprised 4 -6 participants	weekly health professional facilitated sessions followed by 12 x weekly group member facilitated sessions (duration unspecified)	
Marziali, 2009 [50]	Website with email, information, text-based discussion forum, and embedded VC links. VC group for education and psychosocial support	Persons with chronic disease, 60.8	N=18—three groups each with 6 participants	10 x 1 hour weekly health professional facilitated sessions followed by 12 x weekly group member facilitated sessions (duration unspecified)	96
Marziali, 2011 [51]	Education and psychosocial support. Website with email, information, text-based discussion forum, educational	Caregivers, 65.1	N=9—two groups: text-based chat group comprised 40 participants, VC group comprised	10 x 1 hour weekly followed by 10 x 1 hour weekly group member facilitated sessions (duration	20

	videos, and embedded VC links		51, participants split into unspecified no. of subgroups each with 6 participants	unspecified)	
Nyström, 2006 and 2008 [43,44]	Support group (group identified topics for discussion), problem solving	New parents, 20-34	N=9—two groups comprised mothers with 5 participants and fathers with 4 participants	9 x 1-1.5 hours monthly	18
Tsaousides, 2014 [48]	Cognitive behavioral therapy for emotion regulation, education, and skill acquisition.	Persons with chronic disease, 42.8	N=7—two groups comprised 4 and 3 participants	16 x 1 hour twice weekly	16
Wild, 2015 [56]	Focus on education, skill acquisition, and social support.	Persons with obesity, usual care group 41.2, VC group 41.9	N=117—two groups, usual care comprised 56 participants and VC group comprised 58	5 x 90 min face-to-face, 6 x 50 min VC	Approximately 116

			participants		

[a]VC: videoconferencing.

References:

38. Khatri N, Marziali E, Tchernikov I, Shepherd N. Comparing telehealth-based and clinic-based group cognitive behavioral therapy for adults with depression and anxiety: a pilot study. Clin Interv Aging. 2014;9:765-70. doi: 10.2147/cia.s57832. PMID: 24855345.
41. Marziali E, Damianakis T, Donahue P. Internet-Based Clinical Services: Virtual Support Groups for Family Caregivers. J Technol Hum Serv. 2006b;24(2/3):39-54. doi: 10.1300/J017v24n0203. PMID: 23259160.
42. Marziali E, Donahue P. Caring for others: Internet video-conferencing group intervention for family caregivers of older adults with neurodegenerative disease. Gerontologist. 2006a;46(3):398-403. PMID: 16731880.
43. Nyström K, Öhrling K. Parental support: Mothers' experience of electronic encounters. J Telemed Telecare. 2006;12(4):194-7. PMID: 16774701.
44. Nyström K, Öhrling K. Electronic encounters: fathers' experiences of parental support. J Telemed Telecare. 2008;14(2):71-4. doi: 10.1258/jtt.2007.070605. PMID: 31214651.
45. Adamski T, Alfaro MW. Virtual psycho-educative support groups for caregivers of persons diagnosed with dementia. Caring. 2009;28(8):44-6. PMID: 19772023.
46. Austrom MG, Geros KN, Hemmerlein K, McGuire SM, Gao S, Brown SA, et al. Use of a multiparty web based videoconference support group for family caregivers: Innovative practice. Dementia. 2015;14(5):682-90. doi: 10.1177/1471301214544338. PMID: 2015-45450-009.
47. Ehlers DK, Huberty JL, de Vreede G-J. Can an Evidence-Based Book Club Intervention Delivered via a Tablet Computer Improve Physical Activity in Middle-Aged Women? Telemed J E Health. 2015;21(2):125-31. doi: 10.1089/tmj.2013.0360. PMID: 100749832.
48. Tsaousides T, D'Antonio E, Varbanova V, Spielman L. Delivering group treatment via videoconference to individuals with traumatic brain injury: a feasibility study. Neuropsychol Rehabil. 2014;24(5):784-803. doi: 10.1080/09602011.2014.907186. PMID: 24810148.
49. Damianakis T, Tough A, Marziali E, Dawson DR. Therapy Online: A Web-Based Video Support Group for Family Caregivers of Survivors With Traumatic Brain Injury. J Head Trauma Rehabil. 2016;31(4):E12-E20. PMID: 26291634.
50. Marziali E. E-health program for patients with chronic disease. Telemed E Health. 2009;15(2):176-81. PMID: 19292627.
51. Marziali E, Garcia LJ. Dementia caregivers' responses to 2 Internet-based intervention programs. Am J Alzheimers Dis Other Demen. 2011;26(1):36-43. doi: 10.1177/1533317510387586. PMID: 21282276.
52. Lundberg S. The results from a two-year case study of an information and communication technology support system for family caregivers. Dis Rehab Ass Technol. 2014 Jul;9(4):353-8. doi: 10.3109/17483107.2013.814170. PMID: 23855387.
53. Burkow TM, Vognild LK, Østengen G, Johnsen E, Risberg MJ, Bratvold A, et al. Internet-enabled pulmonary rehabilitation and diabetes education in group settings at home: a preliminary study of patient acceptability. BMC Med Inform Decis Mak. 2013;13(1):33. doi: 10.1186/1472-6947-13-33. PMID: 23496829.

54. Burkow TM, Vognild LK, Johnsen E, Risberg MJ, Bratvold A, Breivik E, et al. Comprehensive pulmonary rehabilitation in home-based online groups: a mixed method pilot study in COPD. BMC Res Notes. 2015;8(1):766. doi: 10.1186/s13104-015-1713-8. PMID: 26651831.
55. Banbury A, Parkinson L, Nancarrow S, Dart J, Gray L, Buckley J. Multi-site videoconferencing for home-based education of older people with chronic conditions: the Telehealth Literacy Project. J Telemed Telecare. 2014;20(7):353-9. doi: 10.1177/1357633X14552369. PMID: 99467458.
56. Wild B, Hunnemeyer K, Sauer H, Hain B, Mack I, Schellberg D, et al. A 1-year videoconferencing-based psychoeducational group intervention following bariatric surgery: results of a randomized controlled study. Surg Obes Relat Dis. 2015 Nov-Dec;11(6):1349-60. doi: 10.1016/j.soard.2015.05.018. PMID: 26421929.
