# Supplementary material for: Telehealth Interventions Delivering Home-based Support Group Videoconferencing: Systematic Review
Source: J Med Internet Res. 2018 Feb 2;20(2):e25. doi: 10.2196/jmir.8090 (PMC5816261; doi:10.2196/jmir.8090)
Supplement: Multimedia Appendix 3 [file jmir_v20i2e25_app3.pdf]

Multimedia Appendix 3. Outcome measures and reported findings of included studies.

Author, year, country	Author, year	Assessment of outcomes	Reported findings
Adamski, 2009 [45]	Adamski, 2009	Patient satisfaction and experience	Feasibility: anxiety around using equipment; minor technical difficulties, most of which participants were able to overcome with instructions; IT[a] support by experienced adult trainer.<br><br>Acceptability: adherence rates not reported, most frequently requested intervention day Saturday morning.<br><br>Effectiveness: Quantitative—No figures included but reports both groups experience support and efficacy equally. Qualitative—Accessing group from home highly valued; difficulty in obtaining consent forms sent in the mail.<br><br>Implementation: Redesign of existing face-to-face program.
Austrom, 2015 [46]	Austrom, 2015	Patient satisfaction and experience, caregiver depression, anxiety, burden, self-efficacy, and quality of life	Feasibility: off-the-shelf computers with cable or broadband connection at 200 kbps; IT support via remote computer access or home visits; helpful that research assistant could provide technical support; easy to use equipment.<br><br>Acceptability: one participant dropped out after two sessions, others had an interest in continuing intervention; 80 of 96 sessions attended by remaining 4 participants.<br><br>Effectiveness: quantitative—Trend of improvement in

			caregiver anxiety; 8.0 (standard deviation [SD] 7.3) at baseline to 6.5 (SD 6.1), mean difference 1.5, improved 75%, and depression scores; 8.3 (SD3.6) at baseline to 5.0 (SD 1.4), mean difference 3.3, improved 75%. Difficulties experienced by caregiver increased slightly (mean=1.0). Improvement in self-efficacy score in subgroups, controlling upsetting thoughts and responding to disruptive behavior. *P* values for all scores not reported. Improvement in quality of life for physical health but remained relatively the same for mental health. Qualitative-positive feedback on lack of travel; access to guest speakers; meeting others in similar circumstances.
Banbury, 2014 [55]	Banbury, 2014	Patient satisfaction and experience	Feasibility: connection via high-speed broadband and 4G to tablets or computer; technical difficulties particularly with 4G for rural multi-dwelling homes; IT support via remote access and home visits.<br><br>Acceptability: VC[b] groups highly valued, particularly for meeting new people; adherence to communication protocols; 2 participants dropped out because of technical problems; weekly duration of meetings increased over time; no privacy concerns.<br><br>Effectiveness: qualitative—valued sharing experiences and learning about health literacy and chronic disease self-management an informal group; improved access to group education; those with anxiety found VC less overwhelming than meeting people face-to-face; group cohesiveness especially where group membership was stable.
Burkow, 2013 [53]	Burkow, 2013	Patient satisfaction and	Feasibility: system developed for inexperienced computer users and connected to home TV's; face-to-face training for participants and facilitators.

		experience	Acceptability: high participation rates with no dropouts; requests for longer meetings; interaction and dialogue lack spontaneity; direct communication between peers limited compared with the in-person group.<br><br>Effectiveness: individual consultations perceived as good as face-to-face meetings; exercise deemed a social activity; social aspect considered important, and social support was achieved; chronic obstructive pulmonary disease patients felt meeting by VC conserved energy.<br><br>Implementation: valued individual sessions to ask questions; one comment of VC lacking socialization opportunities.
Burkow, 2015 [54]	Burkow, 2015	Patient satisfaction and experience, usability, quality of life	Feasibility: technology easy to use; mean score of 94.4 out of 100 on usability scale; user manual and training; total IT support time throughout the program was 15 min.<br><br>Acceptability: 80% attended all group and individual sessions; 100% adherence to watching videos before sessions and entering electronic diary data; requests for more group exercise and longer; better communication structure required; protocols for safeguarding privacy in place.<br><br>Effectiveness: quantitative—improvements in quality of life scores although not significant. Qualitative—valued by those who normally have to travel long distances; group cohesion observed; sharing health information related to daily life.
Damianakis	Damianakis	Patient	Feasibility: few technical problems; however,

,<br>2016 [49]	, 2016 [49]	satisfaction and experience, replication of therapeutic group process in VC environment	participants needed reassuring they were not a fault when they did occur.<br><br>Acceptability: attendance, group one=66%, group two=80% of sessions; participants and facilitators adapted readily to communicating; other family members joined ad hoc and were accepted by other participants.<br><br>Effectiveness: qualitative themes consistent with caregiver burden literature and included caregiver-identified issues, enhancing problem-solving strategies, ad psychosocial and self-care needs. Participants reported improved access to needed resources and self-efficacy and acceptance; participant-facilitator interactions paralleled face-to-face support; easy access to support group.<br><br>Implementation: psychotherapeutic group process replicated with regard to cohesiveness, mutual identification, empathetic support and problem-solving strategies.
Ehlers, 2015 [47]	Ehlers, 2015, [47]	Patient satisfaction, physical activity (PA) monitoring and levels, general and physical self-worth, physical activity (PA)	Feasibility: problems with audio delays, background noise, and using time to resolve IT issues; IT tutorial support emailed; varying levels of digital literacy may have affected low participant participation.<br><br>Acceptability: five out of 6 VC participants would have preferred to have met face-to-face citing low social presence; VC group attended fewer meetings with some doing other tasks during sessions.<br><br>Effectiveness: qualitative—both groups reported books

		self-efficacy and self-regulation	helped adopt PA, although there were more improvements by face-to-face group compared with VC group; social support by face-to-face group was valued but lacking with VC group; website accessed mainly at the beginning; blog never accessed by some. Significant decrease in PA planning for VC group (P=.02), whereas face-to-face group improved.
Khatri, 2014 [38]	Khatri, 2014, [38]	Depression	Feasibility: one group had some technical difficulties causing frustration for participants; IT support always online to overcome technical difficulties.<br><br>Acceptability: overall positive response to group VC.<br><br>Effectiveness: quantitative—pre-post intervention scores for the Beck Depression Inventory-II (BDI-II) were comparable across the two delivery formats, with 60% of participants in each group showing a positive change in BDI-II severity classification post intervention, from moderate to low symptoms. Qualitative—both groups bonded and demonstrated group cohesiveness; same therapist for both groups; initial delivery of VC group challenging but became easier after first session.<br><br>Implementation: reliable adherence to the group cognitive behavioral therapy intervention protocol in both delivery formats; themes in group discussions indicated both groups addressed similar issues.
Lundberg, 2014 [52]	Lundberg, 2014 [52]	Patient satisfaction and experience, stress, mental	Feasibility: many technical problems with IT system and technology; continuous need for IT support (provided by call center) and updating system.<br><br>Acceptability: videophone was the most liked function of the IT system; group meetings were an important

		health, service utilization	source of information and enabled people to meet others in similar circumstances and share experiences; however, it is unclear whether or how many of these were face-to-face or by VC.<br><br>Effectiveness: changes were small—no significant reduction in stress or mental health; appreciation of life after intervention was lower than pre study; small increase in self-reported depression; decrease in contentedness; slight increase in happiness, and slight increase in use of services; new social networks were created that served as a self-help group—the main benefit of intervention.
Marziali, 2006a and 2006b [42,41]	Marziali, 2006a and 2006b [42,41]	Patient experience and satisfaction, general health status, depression, activities of daily living, social support	Feasibility: large dropout (n=28); 78% found website easy to use; two IT training sessions provided; website designed for older people; in VC, only the person speaking was visible; manipulating technology was challenging for therapist but eased overtime.<br><br>Acceptability: 95% found experience positive, 5% preferred in-person or telephone contact; VC felt nonintrusive and safe.<br>Effectiveness: quantitative—no differences between control and intervention on any measures; 61% felt sharing experiences via VC was as helpful as in-person. Qualitative—VC provided group cohesion, empathetic communication, improved insight and skills, and helped overcome isolation.<br><br>Implementation: successful replication of face-to-face group process; intervention shifted from structured topic-driven format to more open participants-driven format.

Marziali, 2009 [50]	Marziali, 2009 [50]	Patient satisfaction and experience	Feasibility: 78% felt website easy to use; only active speaker can be viewed; adapted to Internet support group well with little prior technology experience; problems with software and service provider; training provided.<br><br>Acceptability: 95% felt using computers to meet online was positive or moderately positive; liked accessing health care from home, making new friends, and ability to socialize; attendance was good for one group, the other two had a core group who attended regularly.<br><br>Effectiveness: group bonding and cohesiveness in all three groups; online support group cohesion was similar to in-person; group was an important source of social support; reduced sense of isolation.
Marziali, 2011 [51]	Marziali, 2011 [51]	Instrumental activities of daily living (IADL), patient experience neuroticism, self-efficacy, social support, general health, depression, distress, health service use	Feasibility: 95% participants had computers and Internet access but needed assistance installing additional equipment; website easy to access; technical difficulties with VC software.<br><br>Acceptability: text-based chat forum sparsely used; varying feedback on educational videos; VC group provided mutual help and support and forum for sharing information; accessing group from home enabled people to be more open; VC group attended 70% of facilitated sessions, 50% of self-help group facilitated sessions.<br><br>Effectiveness: quantitative—both groups, significant improvement in self-efficacy (*P*=.04), no changes in utilization of health and social services for either care recipient or caregiver; significant differences in

			between-group analyses, the VC group showed greater improvement in metal health (*P*=.02), lower distress scores associated with managing the care recipient's deterioration in mental (cognitive) function (*P*=.02). The chat group compared with VC group had lower distress scores associated with managing IADL (*P*=.02). Regression analysis of three variables (change in personality, self-efficacy, and social support and caregiver distress scores) to change in five variables (5 caregiver distress scores, caregiver physical and mental health) showed no significant changes for chat group. For VC group, these contributed to changes in two caregiver distress domains: distress related to coping with care recipient's mental (cognitive) function (personality *P*=.03, self-efficacy *P*<.001, social support *P*<.001), and distress in helping care recipient manage activities of daily living (personality *P*=.02, social support *P*<.001). Qualitative—chat group reported much less mutual help and support and new knowledge and skills compared with VC group; discussion themes paralleled previous face-to-face groups.<br><br>Implementation: therapist demonstrated consistent adherence to the treatment manual.
Nyström, 2006 and 2008 [43,44]	Nyström, 2006 and 2008 [43,44]	Patient satisfaction and experience	Feasibility: technology was considered fun; sound-led problems led to frustration; picture too small to see body language; men had more positive attitudes than women toward computer use; in-home context affected ability to concentrate because of distractions.<br><br>Acceptability: meetings enjoyable and a feeling of excitement to take part; a good tool to meet new people particularly in rural areas; one group changed meeting

			times to the evening to overcome distractions.<br><br>Effectiveness: for mothers—feeling supported thereby reducing anxiety, improved self-efficacy, reducing isolation and loneliness. For fathers—valued talking with others about things they do not dare to normally talk about. Discussion focus indicated gender differences, with men more problem-focused and women more emotion-focused; all like content driven by members.<br><br>Implementation: confidentiality discussed at initial meeting; the nurse was important to facilitate conversation and overcome initial nervousness; men needed more guidance than women to generate discussion.
Tsaousides, 2014 [48]	Tsaousides, 2014 [48]	Patient satisfaction, emotion regulation, problem solving, knowledge and skills development, remote assessment	Feasibility: minimal problems with technology; email link for VC group to install software; 95.2% used technology with ease.<br><br>Acceptability: 93% satisfaction with quality of treatment; 93.8% attendance; number and length of sessions too short; homework completed 93% of the time; therapist rated full participation 79.5% of time; some felt constrained or needed time to adjust to an online experience.<br><br>Effectiveness: quantitative—high satisfaction with treatment and delivery (66.9 out of 72); no significant differences in outcomes for emotion regulation or problem solving, Therapist rated all participants exceptional or good at skill acquisition, and 6 participants exceptional at generalization of skills.

			Qualitative—positive social experience; emotional regulation skills relevant and useful; beneficial for those who would not have met in-person.
Wild, 2015 [56]	Wild, 2015 [56]	Weight, health-related quality of life (HRQOL), self-efficacy, depression, and eating behavior	Feasibility: dealing with technical difficulties were time consuming.<br><br>Acceptability: low dropout rate (n=9); developed rules and guidelines for delivering group sessions.<br><br>Effectiveness: mean weight loss for all patients was 45.9 kg (standard deviation 16.4) 1 year after surgery; intention-to-treat analyses, no differences between groups in weight loss, excessive weight loss, HRQOL, self-efficacy, eating psychopathology, and depressive symptoms between groups. For VC group, those with clinically significant depression symptoms at baseline (n=29) had significantly better HRQOL (*P*=.03), lower depression scores (*P*=.02). Qualitative—VC enabled good coherence, session structure, and ability to share information and spirit of attendees established fellowship and may have influenced the outcome for those with depression.<br><br>Implementation: general rules and session structure with opportunities share to physical and mental health status, problems and needs and greet and say goodbye.

[a]IT: information technology.

[b]VC: videoconferencing.

References:

38. Khatri N, Marziali E, Tchernikov I, Shepherd N. Comparing telehealth-based and clinic-based group cognitive behavioral therapy for adults with depression and anxiety: a pilot study. Clin Interv Aging. 2014;9:765-70. doi: 10.2147/cia.s57832. PMID: 24855345.

41. Marziali E, Damianakis T, Donahue P. Internet-Based Clinical Services: Virtual Support Groups for Family Caregivers. J Technol Hum Serv. 2006b;24(2/3):39-54. doi: 10.1300/J017v24n0203. PMID: 23259160.
42. Marziali E, Donahue P. Caring for others: Internet video-conferencing group intervention for family caregivers of older adults with neurodegenerative disease. Gerontologist. 2006a;46(3):398-403. PMID: 16731880.
43. Nyström K, Öhrling K. Parental support: Mothers' experience of electronic encounters. J Telemed Telecare. 2006;12(4):194-7. PMID: 16774701.
44. Nyström K, Öhrling K. Electronic encounters: fathers' experiences of parental support. J Telemed Telecare. 2008;14(2):71-4. doi: 10.1258/jtt.2007.070605. PMID: 31214651.
45. Adamski T, Alfaro MW. Virtual psycho-educative support groups for caregivers of persons diagnosed with dementia. Caring. 2009;28(8):44-6. PMID: 19772023.
46. Austrom MG, Geros KN, Hemmerlein K, McGuire SM, Gao S, Brown SA, et al. Use of a multiparty web based videoconference support group for family caregivers: Innovative practice. Dementia. 2015;14(5):682-90. doi: 10.1177/1471301214544338. PMID: 2015-45450-009.
47. Ehlers DK, Huberty JL, de Vreede G-J. Can an Evidence-Based Book Club Intervention Delivered via a Tablet Computer Improve Physical Activity in Middle-Aged Women? Telemed J E Health. 2015;21(2):125-31. doi: 10.1089/tmj.2013.0360. PMID: 100749832.
48. Tsaousides T, D'Antonio E, Varbanova V, Spielman L. Delivering group treatment via videoconference to individuals with traumatic brain injury: a feasibility study. Neuropsychol Rehabil. 2014;24(5):784-803. doi: 10.1080/09602011.2014.907186. PMID: 24810148.
49. Damianakis T, Tough A, Marziali E, Dawson DR. Therapy Online: A Web-Based Video Support Group for Family Caregivers of Survivors With Traumatic Brain Injury. J Head Trauma Rehabil. 2016;31(4):E12-E20. PMID: 26291634.

50. Marziali E. E-health program for patients with chronic disease. Telemed E Health. 2009;15(2):176-81. PMID: 19292627.
51. Marziali E, Garcia LJ. Dementia caregivers' responses to 2 Internet-based intervention programs. Am J Alzheimers Dis Other Demen. 2011;26(1):36-43. doi: 10.1177/1533317510387586. PMID: 21282276.
52. Lundberg S. The results from a two-year case study of an information and communication technology support system for family caregivers. Dis Rehab Ass Technol. 2014 Jul;9(4):353-8. doi: 10.3109/17483107.2013.814170. PMID: 23855387.
53. Burkow TM, Vognild LK, Østengen G, Johnsen E, Risberg MJ, Bratvold A, et al. Internet-enabled pulmonary rehabilitation and diabetes education in group settings at home: a preliminary study of patient acceptability. BMC Med Inform Decis Mak. 2013;13(1):33. doi: 10.1186/1472-6947-13-33. PMID: 23496829.
54. Burkow TM, Vognild LK, Johnsen E, Risberg MJ, Bratvold A, Breivik E, et al. Comprehensive pulmonary rehabilitation in home-based online groups: a mixed method pilot study in COPD. BMC Res Notes. 2015;8(1):766. doi: 10.1186/s13104-015-1713-8. PMID: 26651831.
55. Banbury A, Parkinson L, Nancarrow S, Dart J, Gray L, Buckley J. Multi-site videoconferencing for home-based education of older people with chronic conditions: the Telehealth Literacy Project. J Telemed Telecare. 2014;20(7):353-9. doi: 10.1177/1357633X14552369. PMID: 99467458.

56. Wild B, Hunnemeyer K, Sauer H, Hain B, Mack I, Schellberg D, et al. A 1-year videoconferencing-based psychoeducational group intervention following bariatric surgery: results of a randomized controlled study. Surg Obes Relat Dis. 2015 Nov-Dec;11(6):1349-60. doi: 10.1016/j.soard.2015.05.018. PMID: 26421929.
